# Haematological and hepatic adverse effects of ceftriaxone in ambulatory care: a dual-centre retrospective observational analysis of standard vs high dose

**DOI:** 10.1186/s12879-022-07925-y

**Published:** 2022-12-24

**Authors:** Rakhee Mistry, Timothy M. Rawson, Oliver Troise, Nabeela Mughal, Luke S. P. Moore, Stephen Hughes

**Affiliations:** 1 Chelsea and Westminster NHS Foundation Trust, 369 Fulham Road, London, SW10 9NH UK; 2 North West London Pathology, Imperial College Healthcare NHS Trust, Fulham Palace Road, London, W6 8RF UK; 3 National Institute for Health Research Health Protection Research Unit in Healthcare Associated Infections and Antimicrobial Resistance, Imperial College London, Hammersmith Campus, Du Cane Road, London, W12 0NN UK

**Keywords:** OPAT, Antimicrobial, Antimicrobial stewardship

## Abstract

**Background:**

European Committee on Antimicrobial Susceptibility Testing (EUCAST) breakpoint criteria for methicillin-susceptible *Staphylococcus aureus* (MSSA) treatment with ceftriaxone are based upon high dose (4 g/day) rather than standard dose (2 g/day) posology. This is particularly relevant for invasive infections, and for patients managed via Outpatient Parenteral Antimicrobial Therapy (OPAT), but may result in increased drug toxicity. We quantified the incidence of neutropenia, thrombocytopenia and raised liver enzymes between standard and high dose ceftriaxone in adult patients.

**Method:**

Adult outpatients prescribed ≥ 7 days of ceftriaxone therapy were identified, and clinical, pharmacological, and laboratory parameters extracted from electronic health records between May 2021 and December 2021. Incidence and median time to haematological and hepto-toxicity were analysed. Univariate odds ratios were calculated for neutrophil count and ALT levels with 95% confidence level and Chi squared/Fisher’s exact test used to identify statistical significance.

**Results:**

Incidence of neutropenia was comparable between both groups; 8/47 (17%) in the 2 g group vs 6/39 (15.4%) in the 4 g group (OR 0.89 (95% CI 0.26–2.63), p > 0.999). Median time to neutropenia was 12 and 17 days in the 2 g and 4 g groups respectively. Thrombocytopenia was observed in 0/47 in the 2 g group compared with 3/39 (7.7%) in the 4 g group (p 0.089). Median time to thrombocytopenia was 7 days in the 4 g group. Elevated liver enzymes did not clearly correlate with ceftriaxone dosing; present in 5/47 (10.6%) and 2/39 (5.1%) for 2 g and 4 g respectively (OR 0.45 (95% CI 0.87–2.36), p 0.448). Treatment cessation due to any adverse effect was similar between both groups 2/47 (4.3%) for 2 g and 3/39 (7.7%) for 4 g (OR 1.86 (95% CI 0.36–10.92), p 0.655).

**Conclusions:**

Increased adverse effects with 4 g (over 2 g) daily dosing of ceftriaxone was not observed in an OPAT population. However absolute development of haematological and liver dyscrasias was appreciable—monitoring of liver function and full blood count in patients receiving prolonged ceftriaxone is indicated irrespective of dosing.

## Background

Ceftriaxone, is commonly used as a parenteral antibacterial to treat a variety of infections [1]. Ceftriaxone provides a broad spectrum of antimicrobial activity encompassing common Gram positive and Gram negative bacteria, has a low allergenic and toxicity profile and favourable tissue penetration for the most common infections [1–3]. This, coupled with the convenience of once daily dosing, means it is commonly used for Outpatient Parenteral Antimicrobial Therapy (OPAT).

The licensed dose of ceftriaxone in the UK is 2–4 g daily for adults and children ≥ 12 years of age with a weight ≥ 50 kg [4]. The European Committee on Antimicrobial Susceptibility Testing (EUCAST) breakpoint criteria for methicillin-susceptible *Staphylococcus aureus* (MSSA) treatment with ceftriaxone is based upon high dose (4 g/day) rather than standard dose (2 g/day) [5, 6]. This may (consequently) increase the risk of adverse effects especially for those on prolonged courses via OPAT services.

Adverse effects commonly reported with ceftriaxone use include neutropenia, thrombocytopenia and raised liver enzymes as per UK product licence [3, 4]. Descriptive toxicity studies of ceftriaxone are limited to case studies and those involving special patient groups [7]. Duncan et al. in 2012 conducted a descriptive study of the use of ceftriaxone in outpatient settings and found a prevalence rate of 3.7% (51/1377) of adverse effects associated with ceftriaxone use over a 10 year period but the authors of the study did not differentiate between dosing regimens.

This study aimed to compare the incidence of neutropenia, thrombocytopenia and raised liver enzymes between 2 and 4 g daily dosing of ceftriaxone in patients enrolled in OPAT services.

## Methods

### Study design

A dual-centre retrospective observational study was conducted using an electronic database of outpatients aged ≥ 18 years who were prescribed courses of ceftriaxone for ≥ 7 days at Chelsea and Westminster Hospital and West Middlesex Hospital (London, UK) over a 7 month period (May 2021–December 2021). This time frame was fixed by the local antimicrobial stewardship team to complete data collection as a registered service evaluation. Patients who were prescribed courses < 7 days were excluded because full blood count (FBC), urea and electrolytes (U&Es) and liver function tests (LFTs) were monitored weekly on the service. The study was limited to patients prescribed ceftriaxone in the out-patient setting due to standardised monitoring of patients and the more clinically stable nature of patients treated. Ceftriaxone was administered as 4 g Q24h or 2 g Q24h depending on the treatment indication. 4 g Q24h was limited to patients with confirmed or highly probable MSSA and/or central nervous system infection. 2 g Q24h was used for all other indications.

### Data collection

Patients were identified using local electronic databases (Millennium®, Cerner Corp, USA, and ICNet®, Baxter, UK). Electronic health records were interrogated for demographic data, antimicrobial prescribing data, microbiology results, concurrent antimicrobials, serum biochemistry and haematology results. Information regarding pre-established comorbidities and start and end date of therapy were extracted from electronic health records. Antibacterials prescribed prior to OPAT referral (including those received as an in-patient pre-discharge) were not included in this analysis. Non-antimicrobial medication prescribed concurrently to ceftriaxone therapy were not included in this analysis.

Neutrophil count, platelet count, alanine aminotransferase (ALT) level and alkaline phosphatase (ALP) levels were collected at baseline and after each OPAT review (once weekly) until discharge or cessation of ceftriaxone treatment. Patient follow-up post cessation of therapy was not routinely collected in practice and thus interval-outcomes were not conducted as part of this service review.

### Definitions

As described by the local clinical biochemistry laboratory; neutropenia was defined as a neutrophil count ≤ 2.0 × 10^9^/L and thrombocytopenia was defined as a platelet count ≤ 130 × 10^9^/L. Clinically significant neutropenia and thrombocytopenia were defined as ≤ 1.0 × 10^9^/L and ≤ 50 × 10^9^/L respectively. Raised ALT and ALP levels were defined as three times the upper limit of normal range by the local clinical biochemistry laboratory (where upper limit of normal range are 34 IU/L and 130 IU/L respectively).

When reviewing comorbidities for baseline characteristics, pre-established renal disease was defined as chronic kidney disease stage 3b-5 (eGFR ≤ 44 ml/min), pre-established liver disease was defined as chronic hepatitis, cirrhosis (Child–Pugh score B-C) or carcinoma and pre-established haematological disease was defined as pre-existing leukaemia or other malignant conditions and idiopathic thrombocytopenic purpura.

### Statistical analysis

Median and interquartile ranges were used to describe and compare patient age and duration of therapy. Univariate analysis on non-parametric data was performed by the two-tailed Mann Whitney U test to evaluate continuous variables (age and duration). Categorical data were analysed using the Chi squared/Fisher’s exact test as appropriate. Univariate odds ratios were calculated for neutrophil and platelet counts, ALT and ALP levels with 95% confidence level and Chi squared/Fisher’s exact test used to identify statistical significance. Data were recorded in Microsoft Excel (version 15.0.5459.1000, 2022, Microsoft Corporation, Redmond, WA USA). GraphPad Prism 9.3.1 software (GraphPad Prism, San Diego, CA, USA) was used to complete the univariate analysis.

## Results

### Patient cohort

**Fig. 1:**
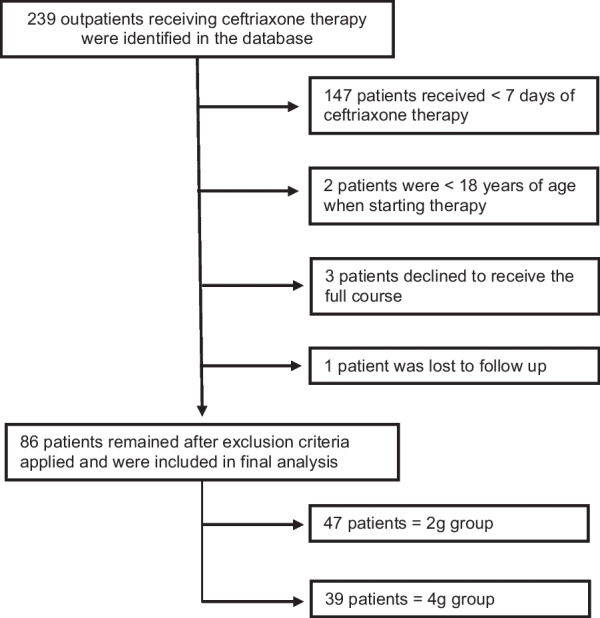
A flowchart summarising the patient exclusion process by which the final patient cohort was selected

239 adult patients prescribed ceftriaxone under the OPAT service were identified during the study period (May 2021–December 2021). Once the exclusion criteria was applied (Fig. 1), a total of 86 patients were included in the final data analysis.

### Baseline characteristics

Baseline characteristics of the study patients treated with 2 g and 4 g ceftriaxone daily dosing is presented in Table 1. There was no significant difference between the 2 g/day and 4 g/day groups across age, gender and duration of ceftriaxone OPAT therapy. Concomitant renal disease was more frequently observed in the 4 g daily dosing group (4/39 (10.2%) versus 0/47 (0%) in the 2 g daily dosing group (p 0.039). No statistical difference in pre-established liver or haematological disease was seen between the two groups.

**Table 1:** A summary of the baseline characteristics of total patient cohort (n = 86) included in the ceftriaxone 2 g and 4 g daily dosing groups

Clinical parameter	2 g (n = 47)	4 g (n = 39)	P value (< 0.05, 95% CI)
Age (years), median (IQR)	54.5 (69.75–40.25)	54.5 (68.25–25.5)	0.354
Gender (female)	21 (44.7%)	14 (35.9%)	0.509
Duration of therapy (days) of OPAT, median (IQR)	19 (33.75–14)	20 (34.25–12.75)	0.103
Co-morbidities			
Pre-established renal disease	0	4 (10.2%)	0.039
Pre-established liver disease	1 (2.1%)	0	> 0.999
Pre-established haematological disease	1 (2.1%)	0	> 0.999
Microbiology			
MSSA (mono-microbial)	2 (4.3%)	18 (46.15%)	< 0.0001
Other Staphylococcal species	2 (4.3%)	1 (2.6%)	> 0.999
Streptococcal species	8 (17%)	2 (5.1%)	0.104
Enterobacterales	2 (4.3%)	0	0.498
Poly-microbial	0	7 (17.9%)	0.003
Anaerobes	4 (8.5%)	0	0.123
Other	1 (2.1%)	0	> 0.999
Nil identified	28 (59.6%)	11 (28.2%)	0.005
Indication			
Bone and joint infections	15 (31.9%)	26 (66.7%)	0.002
Spinal nd central nervous system infections	2 (4.3%)	5 (12.8%)	0.237
Skin and soft tissue infection	2 (4.3%)	6 (15.4%)	0.133
Intra-abdominal infections	9 (19.1%)	1 (2.6%)	0.019
Gynaecological infections	8 (17.0%)	0	0.007
Complex respiratory infections	3 (6.4%)	1 (2.6%)	0.623
Infective endocarditis	3 (6.4%)	0	0.248
Liver abscess	1 (2.1%)	0	> 0.999
Complex urinary tract infection	2 (4.3%)	0	0.499
Pyrexia of unknown origin	2 (4.3%)	0	0.499
Concurrent antimicrobials			
Metronidazole	25 (53.2%)	11 (28.2%)	0.023
Rifampicin	0	3 (7.7%)	0.089
Clindamycin	0	2 (5.1%)	0.203
Fosfomycin	1 (2.1%)	0	> 0.999
Teicoplanin	1 (2.1%)	0	> 0.999
Doxycycline and metronidazole (combination)	4 (8.5%)	0	0.123
Antivirals	0	1 (2.6%)	> 0.999
Antifungals	0	0	n/a
Nil concurrent antimicrobials	16 (41%)	22 (56.4%)	0.050

Comparison of haematological and hepatotoxic adverse effects between 2 and 4 g ceftriaxone

MSSA mono-microbial infections were more likely to be prescribed 4 g daily (18/39 (46.15%)) compared with the 2 g daily dose (2/47 (2.3%)) (p < 0.0001). Poly-microbial infections were more frequently observed in the 4 g group compared to the 2 g group (7/39 (17.9%) vs 0/47, p 0.003) due to a high proportion of MSSA pathogens isolated in this cohort (6/7 (87.5%)). In empiric therapy (no organisms identified at time of prescribing), the standard 2 g daily dose was utilised (28/47 (59.6%) vs 11/39 (28.2%) in the 2 g and 4 g daily dosing groups respectively; p 0.005).

The indication for ceftriaxone based therapy was also associated with total daily dosing. Patients treated for bone and joint infections were more commonly prescribed a 4 g daily dose (26/39 (66.7%) vs 15/47 (31.9%) treated with 2 g daily (p 0.002). MSSA confirmed infections were higher in this sub-group (13/26 (50%) of 4 g vs 2/15 (13.3%) of 2 g). Conversely, intra-abdominal infections and gynaecological infections more frequently utilised a 2 g daily dosage rather than 4 g (9/47 (19.1%) vs 1/39 (2.6%) p 0.019 and 8/47 (17.0%) vs 0/39 (0%) p 0.007, respectively).

Ceftriaxone monotherapy prescribing (no concurrent antimicrobials) was significantly greater in the 4 g group (22/39 (56.4%) of 4 g vs 16/47 (41%) of 2 g, p 0.050). Metronidazole was the most common antimicrobial prescribed concurrently to ceftriaxone; 25/47 (53.2%) vs 11/39 (28.2%), in the 2 g and 4 g daily dosing groups, respectively (p 0.023).

The incidence of study-defined neutropenia, thrombocytopenia and liver enzyme dysfunction observed in the final patient cohort is presented in Table 2.

**Table 2:** A comparison of the incidence of haematological and hepatic adverse effects between patients prescribed 2 g and 4 g daily of ceftriaxone over a 7 month period

	2 g (n = 47)	4 g (n = 39)	OR	OR 95% CI	p value
Analysis of neutrophil count					
Neutropenic	8 (17%)	6 (15.4%)	0.89	0.26–2.63	> 0.999
Analysis of platelet count					
Thrombocytopenia	0 (0%)	3 (7.7%)	N/A	N/A	0.089
Analysis of ALT level					
ALT > 3 × upper limit	5 (10.6%)	2 (5.1%)	0.45	0.87–2.36	0.448
Analysis of ALP level					
ALP > 3 × upper limit	1 (2.1%)	0	N/A	N/A	> 0.999
Treatment cessation due to adverse effects					
Treatment cessation due to adverse effects	2 (4.3%)	3 (7.7%)	1.86	0.36–10.92	0.655

A description of the odds ratio calculated to determine probability of the adverse effect occurring and the statistical significance of the odds ratio (p < 0.05 for statistical significance)

### Neutropenia associated with ceftriaxone treatment

Incidence of study-defined neutropenia on ceftriaxone based treatment were comparable between both dosing groups (8/47 (17%) in the 2 g group vs 6/39 (15.4%) in the 4 g group; OR 0.89 (95% CI 0.26–2.63), p > 0.999. Clinically significant neutropenia was observed in 1/47 (2.1%) in the 2 g group compared with 2/39 (5.1%) in the 4 g group OR 0.40 (95% CI 0.035– 4.62), p 0.588. Median time to neutropenia was 17 and 12 days in the 4 g and 2 g daily dosing groups, respectively.

### Thrombocytopenia associated with ceftriaxone treatment

Study-defined thrombocytopenia was observed in 3/39 (7.7%) patients in the 4 g group compared with 0/47 patients in the 2 g group (p 0.089). Clinically significant thrombocytopenia was observed in 1/39 (2.6%) in the 4 g group. Combined thrombocytopenia and neutropenia was observed in a single patient who received 4 g daily dosage (1/39 (2.6%)). Median time to thrombocytopenia was 7 days in the 4 g group.

### Increases in liver enzymes associated with ceftriaxone treatment

On-treatment hepatotoxicity was not clearly linked with ceftriaxone treatment. A raised ALT was observed in 5/47 (10.6%) of patients in 2 g group and 2/39 (5.1%) in the 4 g group; OR 0.45 (95% CI 0.87–2.36, p 0.448). Median time to raised ALT was 3 days in the 2 g group and 13.5 days in the 4 g group.

Raised ALP on treatment was observed in a single case in the 2 g group (1/47 (2.1%) versus 0/39 in the 4 g group. This suggests no clear correlation between ALP level and dose in this limited sample size. Time to raised ALP was 4 days in the 2 g group.

### Treatment cessation due to adverse effects

Treatment cessation due to any adverse effect (including non-haematological and non-hepatic effects) was similar in 2 g group (2/47 (4.3%)) and 4 g group (3/39 (7.7%)); OR 1.86 (95% CI 0.36–10.92), p 0.655. Diarrhoea (n = 1) and neutropenia (n = 1) resulted in treatment cessation in the 2 g group. Neutropenia (n = 1), thrombocytopenia (n = 1) and skin rash (n = 1) necessitated cessation of therapy in the 4 g group.

## Discussion

Our retrospective observational study compares the incidence of haematological and hepatic adverse effects between two different dosing strategies (standard 2 g daily and high 4 g daily) for ceftriaxone administered in adult patients in an outpatient setting.

We found no significant correlation between the incidence of neutropenia or thrombocytopenia and the dose of ceftriaxone. Rates of laboratory-defined neutropenia and thrombocytopenia were similar between the two groups. A trend to increased numbers of reported thrombocytopenia were seen with 4 g dosing but this was not significant. A larger sample size may be required to specifically quantify any increased risk with higher dosing. Ceftriaxone-associated haematological toxicity is rare with severe events described in single case studies [8]. Our findings are supported by Duncan et al.. in 2012 who observed a prevalence of haematological toxicity (neutropenia and thrombocytopenia combined) 6/51 (11.8%) in patients prescribed ceftriaxone in an outpatient setting [7]. However, the authors conducted a narrative review of available literature rather than an observed study and definitions of neutropenia and thrombocytopenia were not explicit.

Our study found no clear correlation between elevated ALT and ALP levels and dosing of ceftriaxone. This is in contrast to the retrospective cohort study by Nakaharai et al.. who compared changes in liver enzymes in hospitalised patients receiving ceftriaxone 2 g and 4 g daily in courses > 5 days. They found 6/37 (16.2%) patients in the 4 g group had raised ALT, ALP or bilirubin levels compared with 9/434 (2.1%) patients in the 2 g group of ceftriaxone [9]. However, Nakaharai et al. analysed a larger patient cohort which included critically ill patients, those with severe sepsis/septic shock and acutely unwell, hospitalised patients, unlike our study, which may have contributed to identifying a difference in such a way that our study did not.

Prolonged courses of cephalosporins are associated with increased adverse effects. A recent review identified that cephalosporin therapy is associated with increased risk of developing adverse effects with each day of therapy (OR 1.07, 95% CI 1.01–1.12) (n = 12 studies, 3459 patients) [10]. Onset of neutropenia was found in trial data to occur after approximately 28 days of therapy [3]. We observed a median time to haematological adverse effects much earlier than 28 days. This is likely confounded by the prior exposure to antibacterials pre-discharge to the OPAT clinic in our population. Nakahari et al. observed median time to drug-induced hepatic injury as early as 8 days in the 2 g group and 16 days in the 4 g group which were analogous to our findings [9].

The inconsistency in reported time to adverse effects reiterates the need for continuous and frequent monitoring of patients on systemic antimicrobials such as ceftriaxone, independent of dose used. Locally, weekly monitoring and review in the multi-disciplinary team meeting is completed for all OPAT patients.

### Limitations

Our study was limited by the small number of patients enrolled across this dual-centre OPAT centre. A follow-up study investigating adverse effects of prolonged ceftriaxone and other OPAT-based therapies is required to accurately quantify patient adverse events.

Our study collected biochemical and full blood count data at baseline and at once weekly intervals (each OPAT review) creating the possibility that some changes in liver function and neutrophil and platelet count were missed. Transient changes may occur between monitoring but no long-term toxicities would be expected to occur within the weekly bloods; the clinical significance of any transient changes would therefore be limited.

Baseline characteristics were unequal between the two study groups. The need for high-dose ceftriaxone with MSSA and the associated orthopaedic related infections were expectantly biased for the 4 g daily dosing group. Conversely, intra-abdominal and gynaecological infections were expectantly biased for the 2 g daily dosage due to Gram negative and anaerobic organisms having greater clinical significance. A significantly greater proportion of patients with pre-established renal disease was observed in the 4 g group which may have contributed to the incidence of adverse effects due to this population’s altered pharmacokinetic handling of ceftriaxone and their propensity for comorbidities and concurrent medications.

The final limitation was our inability to include pre-OPAT antimicrobial prescribing in this analysis. The overall burden of ceftriaxone or other systemic antimicrobials has therefore not be quantified. Pre-OPAT exposure to antimicrobials may contribute to the observed toxicities later in treatment. Unlike Nakaharai et al., we have sought to focus on the OPAT population solely to minimise infective related toxicities. This selective group are screened for clinical stability prior to acceptance and thus are more clinically stable than the hospitalised patient group. The time to toxicity may therefore be skewed by prior exposure in this study.

## Conclusions

Guidance from EUCAST on interpretation of per-pathogen breakpoints is set against use of higher 4 g per day of ceftriaxone in infections suspected or confirmed to be caused by MSSA (rather than standard 2 g daily dosing). The hypothesised increase in incidence of adverse effects associated with this higher dosing regimen was not observed in this retrospective study of adult OPAT patients.

Larger studies are needed to ascertain if ceftriaxone-associated adverse effects are dose-dependent. Such data would greatly influence the decision-making and monitoring recommendations from antimicrobial stewardship teams and those working in the OPAT settings. We recommend that clinical teams are mindful of the adverse effects of ceftriaxone and that patients prescribed courses > 7 days are regularly monitored for abnormal liver function and full blood count results.

## Data Availability

The datasets analysed during the current study and further details on gaining access to the intervention reported within this study are available from the first author (RM rakhee.mistry@nhs.net) on reasonable request, as long as this meets local ethics and research governance criteria.

## Abbreviations

ALP: Alkaline phosphatase
ALT: Alanine aminotransferase
CI: Confidence interval
EUCAST: European Committee on Antimicrobial Susceptibility Testing
IQR: Interquartile range
MSSA: Methicillin-susceptible Staphylococcus aureus
OPAT: Outpatient parenteral antimicrobial therapy
OR: Odds ratio
